# On the Path to a New Generation of Cement-Based Composites through the Use of Lignocellulosic Micro/Nanofibers

**DOI:** 10.3390/ma12101584

**Published:** 2019-05-15

**Authors:** Rafel Reixach, Josep Claramunt, M. Àngel Chamorro, Joan Llorens, M. Mercè Pareta, Quim Tarrés, Pere Mutjé, Marc Delgado-Aguilar

**Affiliations:** 1 Department of Architecture and Construction Engineering, University of Girona, 17004 Girona, Spain; rafel.reixach@udg.edu (R.R.); mangel.chamorro@udg.edu (M.À.C.); joan.llorens@udg.edu (J.L.); mm.pareta@udg.edu (M.M.P.); 2 Agri-Food Engineering and Biotechnology Department, Polytechnic University of Catalunya, 08860 Castelldefels, Spain; josep.claramunt@upc.edu; 3 LEPAMAP Research Group, Chemical Engineering Department, University of Girona. C/Maria Aurèlia Capmany, 61, 17003 Girona, Spain; pere.mutje@udg.edu (P.M.); m.delgado@udg.edu (M.D.-A.)

**Keywords:** lignocellulosic micro/nanofibers, cement-based composites, mechanical properties

## Abstract

Due to its high biocompatibility, bio-degradability, and low cost, cellulose finds application in disparate areas of research. Here we focus our attention on the potential applications of cellulose nanofiber in cement-based materials for the building sector. We first describe the chemical/morphological composition of cellulose fibers, their process and treatment, the characterization of cement-based composites, and their flexural strength. In recent research in this field, cellulose has been considered in the form of nano-sized particles, i.e., cellulose nanofibers (CNF) or cellulose nanocrystals (CNC). CNF and CNC are used for several reasons, including their mechanical and thermal properties, their extended surface area and low toxicity. This study presents some potential applications of lignocellulosic micro/nanofibers (LCMNF) in cement-based composites in order to improve flexural strength. Samples were made with 0.5-1.0-1.5-2.0 wt% of LCMNF obtained from pine sawdust, CEM I (Portland) and a 1:3 cement-water ratio. The composites were then tested for flexural strength at 7, 14, and 28 days and the evolution of flexural strength was assessed after water immersion during 72 h. Scanning electron microscopy was employed to visualize the bond between LCMNF and the cement matrix. Results showed that LCMNF improved the flexural strength of the composite in all the dosages used.

## 1. Introduction

In the last few decades, an important number of articles have been published on the use of natural fibers in the manufacture of composite materials. Many of these studies have been focused on the use of plastic matrixes with the addition of natural fibers [1,2,3,4,5], but in recent years some have focused on cement-based matrixes mixed with fibers [6,7]. The interest of the latter is basically for the construction sector where cement-based materials are widely used. The addition of fibers helps the end product to acquire a tensile strength that the cement matrix by itself does not have. These natural fibers replace other traditional materials such as steel, glass, and asbestos [8] which have hitherto been extensively used. This change in trend is due to the added value that natural fibers bring to these materials, especially from the point of view of sustainability. These are fibers with a great strength, especially tensile, as well as being environmentally-friendly and cheap. This latter aspect makes this type of composite ideal for use in developing countries [9,10,11].

Several types of natural fibers have been used for both plastic- and cement-based matrixes, but in recent years the use of cellulose fiber has become increasingly generalized given its greater tensile strength. The fibers used—wood pulp, sisal, Manila hemp or cotton fibers—for cement-based composites have a length of between 2 and 10 mm and a diameter of between 10 and 30 µm [11,12,13,14]. They are used in the form of pulp, since both flexural strength and hardness are considerably increased compared to the use of fibers in filaments. The main problem with the use of natural fibers with cement-based matrixes lies in their durability, since they degrade in contact with cement given that it is an alkaline material [13,15,16,17]. 

The latest step in this process has been the use of cellulose nanofibers. These fibers provide a greater contact surface with the matrix, which improves its adhesion substantially, obtaining a more homogeneous material and, therefore, one with better mechanical properties. These nanofibers have basically been applied in polymer matrixes and their use in cement-based matrixes is very recent [18,19,20,21]. Different authors have distinguished two types of nanocellulose: cellulose nanocrystals and cellulose nanofibrils. The former are obtained by treating the fibers with acids, while the latter are obtained by mechanical disintegration. They have also been produced in some studies by using bacterial cellulose [22,23].

The aim of this study was to evaluate the capacity of lignocellulosic micro/nanofibers (LCMNF) for their use in cement-based materials. For this purpose, we studied the flexural behavior of a Portland cement matrix with the addition of LCMNF obtained from pine wood sawdust, and without the addition of aggregate. Different cement/LCMNF dosages were studied for the same water–cement ratio to evaluate the flexural strength of the dry sample. Subsequently, the same mechanical property was studied after the sample had been subjected to different immersion times. Naturally, the properties of the LCMNF, and the process followed to obtain them, were previously determined. The addition of LCMNF up to a certain percentage improved the flexural strength of the matrix, which was partially lost once subjected to immersion, though remaining at acceptable values. These results are promising and open up a new field of research into cement composites with LCMNF for application in the construction sector. 

## 2. Results

### 2.1. Characterization of the Pulp and LCMNF

Pine sawdust was used as raw material for the production of LCMNF. First, the sawdust was submitted to a digestion process in the presence of NaOH and antraquinone as a catalyst, to be then fibrillated in a Sprout-Waldron refiner and, finally, partially bleached with NaClO. Table 1 shows the morphology and chemical composition of the pulps.

Pine sawdust exhibited a chemical composition similar to the values reported in the bibliography [24]. The lignin content was significantly high, as was the cellulose. With the first treatment, which had a yield of 45.4%, meaning that almost 55% of the original raw material was dissolved by the black liquor, lignin content was decreased to almost half, increasing the relative amount of cellulose. Not only lignin, but also hemicellulose was significantly removed from the sawdust, decreasing its presence from 12.30% to 5.89%. After one hour of bleaching, lignin content had decreased to 11.7% with the subsequent increase in cellulose content. Part of the hemicelluloses was also removed, but to a lesser extent than during the first treatment. The yield of this operation was significantly higher than in the first stage, which accounted for 96.7%. The partially-bleached pulp was used for the production of LCMNF and its characterization can be found in Table 2.

The obtained LCMNF exhibited a yield of fibrillation of 21.03%, meaning that about 79% of the material cannot be considered as nanostructured. This yield of fibrillation was totally in consonance with the obtained transmittance at 800 nm, which accounted for 41.6%. The carboxyl content was of the magnitude of common unbleached fibers, since no oxidation of cellulose was carried out during the mechanical treatment. The most significant effect of the mechanical treatment was found in the cationic demand, which is indicative of the average specific surface. In this case, the specific surface was estimated to be around 88 m^2^/g and, if cellulosic fibers (both micro and nano) are considered as perfect cylinders, an average diameter of 28 nm can be considered. Finally, the degree of polymerization was obtained by means of viscosity measurements, obtaining a value of 307.

### 2.2. Characterization of the Cement-Based Composites

**Figure 1:**
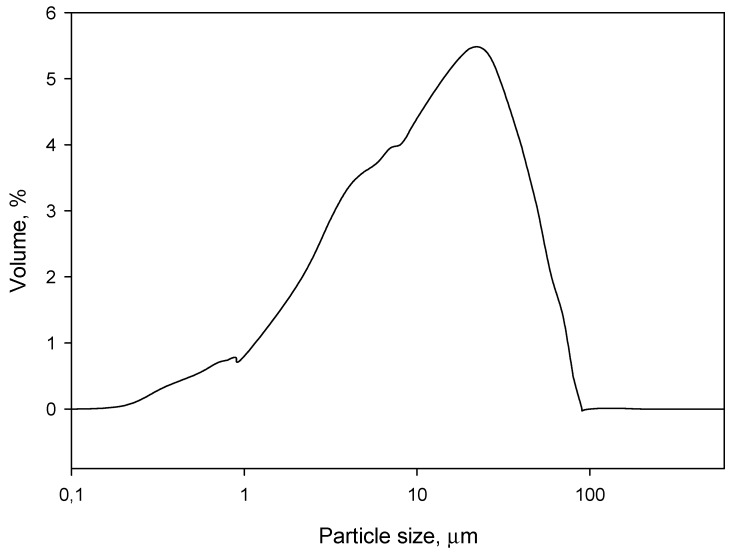
The particle size distribution.

First of all, the particle size distribution was obtained, as reflected in Figure 1. It was found that most of the particles accounted for 20 µm, approximately, while all the particles were below 85 µm.

**Figure 2:**
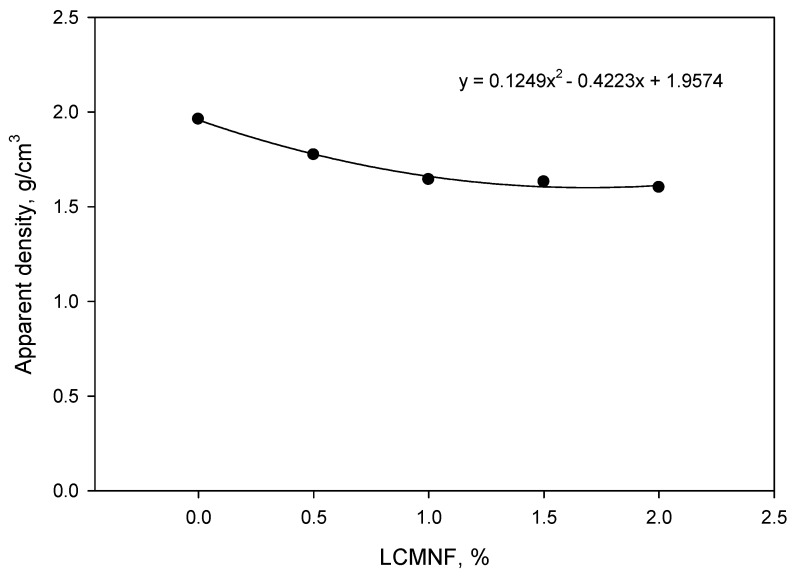
Evolution of apparent density of dried cement-based composites as the amount of LCMNF was increased.

As reported in the Materials and Methods section, different amounts of LCMNF (ranging from 0 to 2 wt%) were incorporated into the cement matrix, together with a constant amount of plasticizer. This amount was not increased due to processability issues, since the viscosity was drastically increased as the amount of LCMNF was increased, together with the limitations of the liquid–solid ratio established at the beginning of experimentation (1:3). In fact, this could be one of the main limiting effects of incorporating fibers with such surface area and water retention capacity. Figure 2 shows the density of the obtained composites.

**Figure 3:**
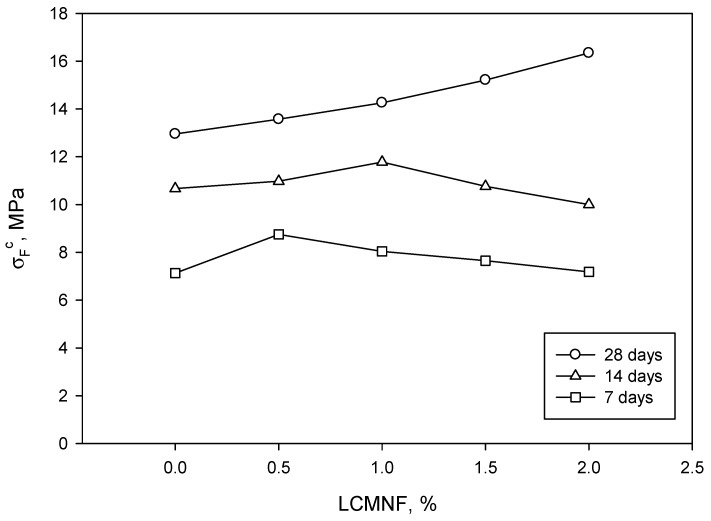
Evolution of flexural strength of the resulting cement-based composites after 7, 14, and 28 days of material curing.

As can be seen, due to the differences between the density of cement and cellulose, lighter materials were obtained as the amount of LCMNF was increased. On the one hand, cellulose presents about the half the specific mass of cement, a fact that can decrease the weight of the resulting composite. On the other, due to the high water-absorption capacity of LCMNF, it is expected that once dispersed into the cement matrix, LCMNF will create voluminous water-based structures that will promote porous structures. In addition, the water content of the specimens is expected to be higher, mainly due to the higher hydrophilic character of cellulose against cement. In fact, as will be later discussed (Figure 3), the water content was higher when the amount of LCMNF was increased. Since the curing process of cement was carried out at room temperature, shrinkage forces on LCMNF were not promoted, avoiding thus further compaction of the structures.

The resulting composites were tested at flexural with the purpose of confirming the role of LCMNF in terms of reinforcing potential. Again, the amount of LCMNF ranged between 0 and 2 wt%, leading to five different materials. Figure 3 shows the results after 7, 14, and 28 days of material curing.

**Figure 4:**
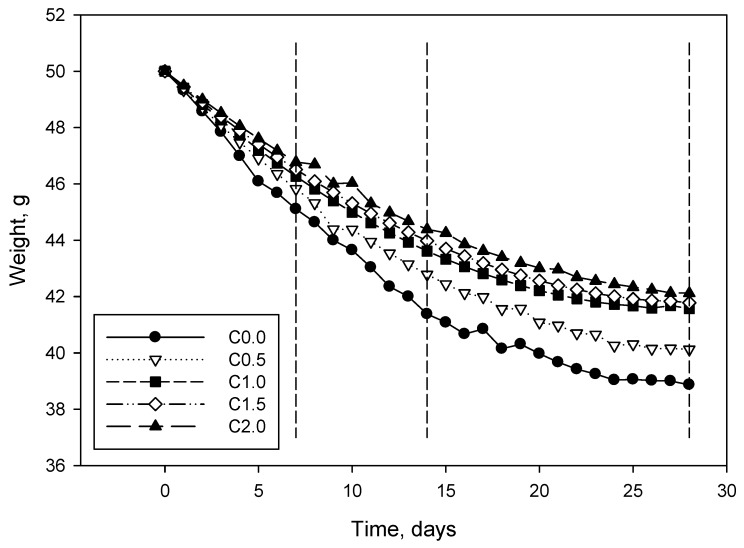
Weight of the different cement-based composites depending on curing time, from 1 to 28 days. The grey and discontinuous vertical lines indicate testing days.

As can be seen, the best performance of the composites was found after 28 days, as per the standard recommends. Although the behavior at 7 and 14 days seems strange, the obtained results were entirely to be expected. Bearing in mind that specimens were produced at a constant water–cement ratio, as the amount of LCMNF was increased, more water was retained and bonded to cellulose, mainly due to hydrogen bonding and van der Waals forces. Considering the different reactions that take place between water and cement during the curing process, it is completely understandable that curing time might be higher as the amount of LCMNF is increased. In fact, cement reacts in water, releasing several ions into the water and, once it is saturated, different salts and oxides precipitate into the cement surface. In this sense, since water is retained by LCMNF, the amount of water free to react with cement is lower, delaying the different reactions that take place during the curing process. Due to the high water-absorption capacity of LCMNF, 7 and 14 days were insufficient to completely dry the cement-based composites. Hence, as the amount of LCMNF was higher, the water content was too. Thus, the presence of water significantly affected the mechanical performance of the composites. After 28 days, the bonding between the cement matrix and LCMNF was, presumably, increased. The neat cement matrix exhibited 7.13 MPa after 7 days curing, a value that was increased to 10.67 after 7 more days (49.09% increase) and further increased after 14 days (28 days from composite preparation) until 12.95 MPa was reached. The incorporation of 2 wt% of LCMNF increased by 26.18% the flexural strength of the cement in the case of the 28-day samples. Thus, analyzing the values for the C2.0 samples, flexural strength increased 39.28% from 7 to 14 days and 63.40% from 14 to 28 days, becoming apparent the role of water content in cement-based materials. Test specimens were weighted every 24 h to further assess the water retention capacity of the composites. Figure 4 shows the evolution of the weight of the different prepared composites, from day 1 to day 28. 

**Figure 5:**
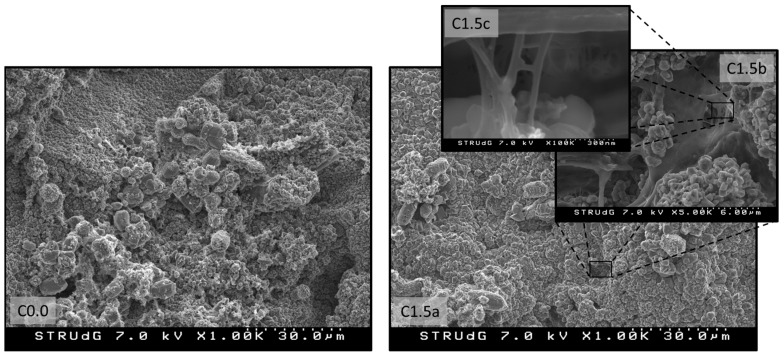
FE-SEM micrographs of (C0.0) neat cement and (C1.5) reinforced cement with 1.5 wt% of LCMNF.

The broken specimens were observed by means of FE-SEM. Two different samples were observed; one containing 1.5 wt% of LCMNF and one without any reinforcement. The distribution of LCMNF within the matrix became apparent, corroborating the results obtained during flexural testing. In fact, the mechanical properties of a composite depend on many factors, such as the intrinsic properties of the reinforcement and the matrix, the interphase between both components and, obviously, the distribution of the reinforcement within the matrix. There is no doubt that the good interaction between LCMNF and cement was assured, mainly due to their hydrophilic character. Accordingly, the distribution of the LCMNF within the matrix was assessed by FE-SEM, as shown in Figure 5.

At first sight, no differences can be observed between the neat cement and the 1.5 wt% LCMNF reinforced composite. Apparently, the same structures can be observed and no significant differences in porosity are shown. However, Figure 5 (C1.5b and C1.5c) shows some nanostructured cellulosic fibers between the cement microstructures, which indicate that more bonds per volume unit are created when using such reinforcements in cement composites. In fact, this explains the higher flexural strength of the composites as the amount of LCMNF is increased. Several zones of the fracture were observed and no broken ends of LCMNF, indicating that the weakest regions of the composites were, in fact, where the presence of LCMNF was residual or null.

**Figure 6:**
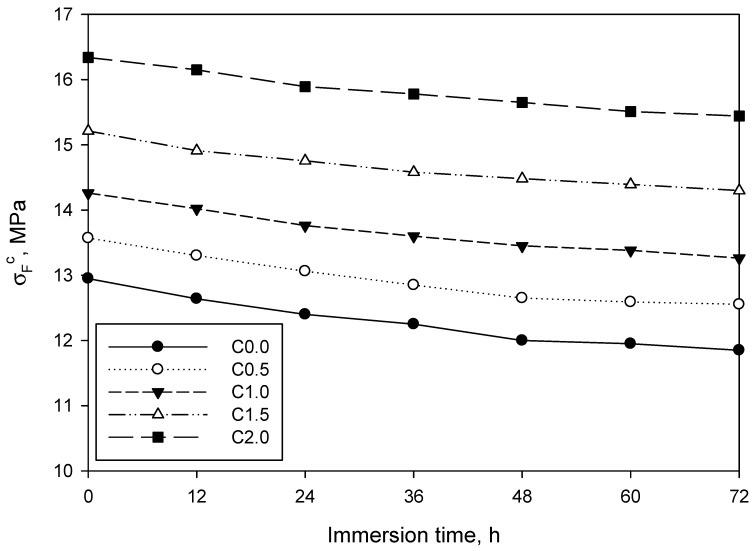
Evolution of flexural strength as immersion time was increased. Tests were performed every 12 h and lines between dots merely connect the data series.

Due to the high hydrophilic character of LCMNF, durability tests were performed as described in the Materials and Methods section, aimed at determining if water immersion could have a direct impact on the mechanical properties of the resulting cement-based composites. Figure 6 shows the evolution of flexural strength as immersion time was increased.

**Figure 7:**
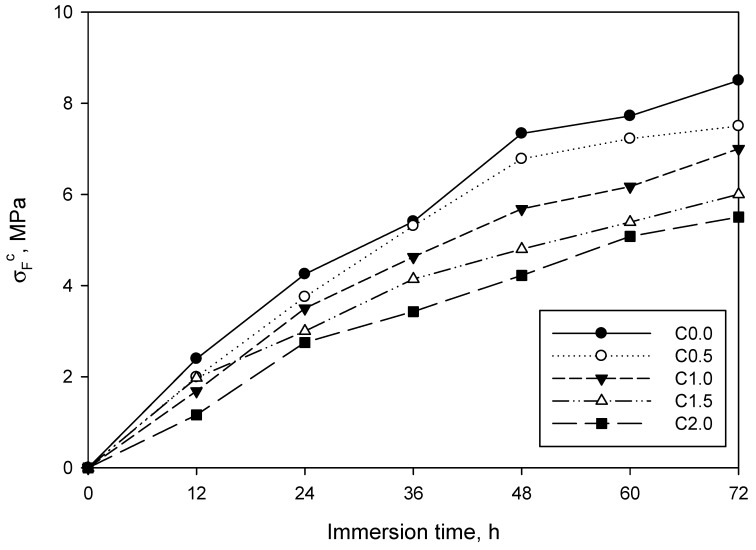
Relative flexural strength loss as a function of immersion time. Values refer to the non-immersed samples.

As shown, flexural strength was affected in different magnitude by water, depending on the LCMNF content. In all cases, the effect of water was more pronounced during the first 24 h than during the second and third day. To further understand this, Figure 7 shows strength loss as immersion time was increased.

Relative flexural strength loss was lower as the amount of LCMNF was increased. In addition, the beginning of a plateau appeared from 48 to 72 h, meaning that the loss in flexural strength was becoming lower. This could be associated to the saturation of cement and LCMNF, which were not able to absorb more water and, consequently, the structure of the composite was not significantly varied from this time. Considering the hydrophilic character of LCMNF, one could expect that the reinforced samples would be the ones further affected by water. Surprisingly, LCMNF appeared to be beneficial for durability, probably due to the presence of lignin in the reinforcement, which conferred a partially hydrophobic character on it.

## 3. Discussion

As noted in the previous section, the flexural strength of the cement /LCMNF composite at 28 days curing grew linearly as LCNMF were added. Maximum flexural strength, 16 MPa, was obtained with 2% of LCMNF. Therefore, would 2% of LCMNF be the percentage that provided the best flexural result? We were not able to confirm this since flexural testing was not conducted with higher percentages as some researchers have set the limit at which the composite does not lose its mechanical properties at 2% [25]. These results came close to, and even slightly improved, those obtained from sisal cellulose nanofibers, where a flexural strength of 14 MPa was reached in the case of mortar. The increase in the flexural strength of mortars manufactured with nanofibers compared to those made with cellulose fibers was 40% [18]. What does become clear is that the use of nanofibers substantially improves flexural strength with respect to the use of vegetable fibers. When palm leaf fibers are used, a flexural strength of between 9.4 and 9.9 MPa is obtained; with coconut shells, it is between 4.26 and 4.68 MPa; with palm kernel shells, it is between 2.75 and 4.00 MPa, and with hemp fibers, it is between 3.10 and 5.18 MPa. Microcellulose fibers provide the highest values, between 6 and 12 MPa, not far from the 16 MPa obtained in our study. Prior treatment of the fibers before their incorporation in the cement-based matrix helps to slightly increase—between 10 and 30%—their flexural strength [26]. 

It is interesting to evaluate the behavior of the fibers before the total hardening of the material at 28 days, as we have already done in the previous section. In this study, a very low water–cement ratio (0.33) was used. Some research studies mention that the water–cement ratio necessary to achieve the total hydration of Portland cement, depending on its composition and fineness, is between 0.21 and 0.33. With this amount of water, there are not enough spaces created for hydration products to be deployed, so water–cement ratios in the order of 0.36 a 0.38 have to be used [27]. In more recent studies, a minimum stoichiometric water–cement ratio of 0.23 has been considered necessary for the hydration of cement [28]. As we can see, we were above the stoichiometric water–cement ratio, but below the optimal ratio for the correct hydration of cement paste. This could mean that the low water–cement ratio was not enough to completely hydrate the cement. Analyzing the literature in this regard, we can see that there are cement composites, such as C_2_S (belite) and C_3_S (alite) that have reacted in 30 and 70%, respectively, after 28 days, and continued to react for more than a year later. These two components were responsible for binding the material through the formation of hydrated calcium silicates [29,30]. Thus, if the cement was hydrated, the decrease in flexural strength at 7 days of 0.5% of LCMNF, and at 14 days of 1% of LCMNF (Figure 2) could be attributed to the large surface area of LCMNF, which hindered adherence in the first days of the hardening of the cement. Nonetheless, further experimentation should be performed to understand the dependence between surface area of different fillers and hardening of cement. Initially, the cement did not have sufficient flexural strength, whereas the fibers did, so when they were stretched, a phenomenon known as fiber pull-out occurred. Thus, it became clear that the higher the percentage of LCMNF, the less flexural strength the composite had. This behavior resembled that of type I/II cement paste with cellulose nanocrystals in which flexural strength increased compared to the sample without fibers in the first 3 days with low fiber content (0.2%) and decreased linearly when the percentage was increased by up to 1%. In the case of flexural strength at 7 days, it increased when fiber content was 0.5% and decreased for higher fiber contents. This behavior is attributed to a delay in the composite setting in the first few days due to water not being able to find its way to hydrate the cement grains, probably because of sugars in the paste [31]. This effect can be mitigated by using additives that accelerate the carbonation of the matrix composites during curing [20,32]. Several authors have suggested that nanofibers facilitate a strong bond between these and the matrix, leading to the fragility of the composite, while microfibers confer a plastic behavior on it [18,21]. However, the high water-retention capacity of LCMNF could reduce the hydration of cement, thereby delaying the cement curing process over time. It could, therefore, account for why properties decrease a few days before curing completion as the percentage of LCMNF increase, whereas this affect is not observed after 28 days.

The immersion in water of manufactured composites, which continues to be a test of their durability, was carried out by successively immersing the samples in water for 72 h. No substantial differences were observed between the flexural behavior of the sample with fibers and the sample without. If we look closely at Figure 5, the straight lines of the five manufactured composites behave in a similar way, being parallel to each other. Naturally, if flexural strength is analyzed in relative terms, the fewer fibers the composite has, the greater the loss is. This loss of flexural strength adjusts in a similar way to the behavior of similar composites which have been studied by other researchers, despite a few exceptions. In hybrid mortars to which 8 wt% of bamboo pulp and 1 wt% of nanocrystals had been added, a similar flexural strength (20 MPa) was observed at 28 days, and after undergoing 200 immersion and drying cycles. These same composites without cellulose nanocrystals, with 9% bamboo pulp, gained about 4 MPa of flexural strength with respect to the control specimen, going from 13 to 17 MPa [20].

Future research should focus on a thorough analysis of the cement hydration process when LCMNF is added, as has already been done for nanocrystals [33]. In other words, it should analyze, as with aggregate used in cement paste, if the water contained in LCMNF facilitates cement hydration, or to the contrary, is absorbed by them, since these hydrophiles are part of the water needed by cement for its correct hydration. Some authors have pointed out that during the hydration process, part of the water in the fibers is transferred to the cement paste to favor the total hydration of the composite [20,33]. This is no trivial matter as the water–cement ratio plays a fundamental role in the strength of the composite, be it compressive, tensile or flexural, since all these mechanical characteristics are interrelated. It is also an important factor for achieving the workability of the material. 

## 4. Materials and Methods

### 4.1. Materials

Pine sawdust was used as a raw material for the production of LCMNF and it was kindly provided by Mas Clarà de Domeny S.L. (Girona, Spain). The selected cement was cement CEM I Portland and it was provided by Cimencat S.A. (Riudellots de la Selva, Spain). ViscoCrete 3425 from Sika AG (Baar, Switzerland) was used as a superplasticizer for concrete in order to promote LCMNF dispersion within the cement matrix. All the reagents for LCMNF production and characterization were supplied by Sigma Aldrich Co (Barcelona, Spain).

### 4.2. Chemi-Thermomechanical Pulp Production and Characterization

First, pine sawdust was further milled and sieved through a 1 mm mesh with the purpose of assuring homogeneity during pulping. Then, the sawdust was introduced in a hermetic rotary digester (LEPAMAP, Girona, Spain) equipped with a heating system. The solid-liquid ratio was 1:6 and thermal treatment was conducted in the presence of 10 wt% of NaOH and 0.1 wt% of antraquinone as a catalyst, at 180 °C for 90 min. Finally, the valve was gradually opened until atmospheric pressure was reached. At this stage, pulp yield was determined as follows:

(1) $$Yield=\frac{m_{f}}{m_{i}}\cdot 100,$$

where *m_f_* is the final dry weight and *m_i_* is the weight of the dry sawdust introduced.

After digestion, the treated sawdust was passed through a Sprout-Waldron mechanical refiner (Muncy, PA, USA) until constant fiber diameter. The morphological analysis of the pulp was performed by means of a MorFi Compact Fiber & Shive Analyzer from Techpap (Gières, France).

Finally, the obtained pulp was bleached for one hour in a digester at 70 °C and 8 wt% of NaClO with regard to the amount of dry pulp, as reported elsewhere [34]. Yield was also calculated after this stage.

Chemical composition, raw material and pulps, were determined as described by Tarrés et al. (2017) [35] and the corresponding standards for each component. Extractive fraction, Klason lignin and Kappa number were determined according to standards T222 om-15 and ISO 302:2015. Hemicellulose content was determined by means of high-performance anion exchange chromatography (HPAEC) and cellulose, by difference from 100% [36].

### 4.3. Production of LCMNF

The partially bleached pine sawdust pulp was first subjected to a refining process in a PFI mill (NPFI02) from IDM (San Sebastian, Spain) at 20,000 revolutions. The consistency at this stage was set at 10 wt% due to the equipment requirements. Then, water was added until reaching a consistency of 2 wt% and passed several times at different pressures through a high-pressure homogenizer PANDA PLUS 2000 from Gea Niro Soavi (Parma, Italy). This stage was carried out following the sequence of 3 passes at 300 bar, 3 passes at 600 bar and 3 passes at 900 bar. Due to composites manufacturing requirements, LCMNF suspensions were concentrated in an oven until achieving a consistency of 8 wt%.

### 4.4. Characterization of LCMNF

The characterization of the obtained LCMNF was conducted according to the methodology described in a previous study [37]. Cationic demand of the LCMNF suspension, understood as the amount of standard cationic polymer required to neutralize the highly anionic surface of the LCMNF, was measured by means of a Mütek PCD 04 particle charge detector from BTG International Ltd (London, UK). First of all, LCMNF were dispersed in a poly-DADMAC solution at 0.1 wt% and stirred for 10 min. Then, the suspension was centrifuged to promote LCMNF precipitation. The supernatant was then titrated with the anionic standard polymer Pes-Na. Both standard polymers were acquired from BTG. Carboxyl content was determined by means of ionic exchange between two defined pHs, 2.8 and 10. This methodology is based on the ionic exchange that occurs between carboxylic groups from cellulose and zinc cations from an aqueous suspension. The first step was to demineralize the LCMNF through the addition of 62.5 mL of Zn(O_2_CCH_3_)_2_ per gram of fiber at pH 1. Then, the suspension was kept under stirring for 30 min and then washed both with Zn(O_2_CCH_3_)_2_ and distilled water. After this stage, 250 mL of Zn(O_2_CCH_3_)_2_ at pH 7 were added and filtered. The filtrate was titrated with an EDTA solution at 0.1 M using black eriochrome as an indicator. This titration was performed at pH 10.

Both cationic demand and carboxyl content can be used to estimate the specific surface and average diameter of LCMNF. This methodology is based on the assumption that the interaction between the LCMNF surface and the added poly-DADMAC during cationic demand determination occurred through two different mechanisms: on the one hand, part of the polymer was retained by ionic interactions between COO^−^ groups from the LCMNF surface and part of the cationic polymer. On the other, the rest of poly-DADMAC was retained by hydrogen bonding and Van der Waals forces. This assumption can only be made if the poly-DADMAC used had a high molecular weight, which was the case [38]. 

Another relevant parameter of LCMNF is the yield of fibrillation. This was determined by centrifugation at 4500 rpm for 20 min of a 0.1 wt% LCMNF suspension. This process allows those LCMNF with diameters in the nano domain (in the supernatant) to be separated from those that are bigger. The relationship between the nanofibrillated part and the total initial dry weight is the yield of fibrillation. The degree of polymerization was determined by means of intrinsic viscosity measurements [39]. Finally, the transmittance of a 0.1 wt% LCMNF aqueous suspension was determined using a Shimadzu UV-160A UV-Vis spectrophotometer (Kioto, Japan). 

### 4.5. Preparation of the Cement-Based Composites

Composites were prepared in a Brabender mixer (Duisburg, Germany) to ensure a good dispersion of the LCMNF into the cement matrix. The solid-liquid ratio was set at 3:1 and plasticizer was added as extra additive (1 wt% with regard to the total weight). This solid-liquid ratio was selected to have the best mechanical performance after 28 days curing of the resulting composites, as has been reported before. The amount of LCMNF was calculated to have composites containing 0, 0.5, 1.0, 1.5 and 2.0 wt% of reinforcement. Depending on the amount of LCMNF, the amount of extra water was modified within the limits of the abovementioned solid-liquid ratio. Table 3 shows the experimental batch for the development of this work, as well as the amount of each material to produce a batch in the Brabender mixer (50 g).

The amount of plasticizer was set at 1 wt% to have 0.20 N·m of torque during the preparation of composites, which was monitored throughout the entire process. This torque ensured a good dispersion of LCMNF into the cement matrix. Depending on the purpose, different specimens with different dimensions were prepared, using specific molds for each characterization. 

### 4.6. Characterization of the Cement-Based Composites

First of all, the particle size distribution of cement was determined by means of a Mastersizer 2000 (Malvern Instruments, Ltd., Malvern, UK).

Cement composites were characterized in terms of physical and mechanical properties and durability. Apparent density was determined according to UNE-EN 1015-10 standard, which consists of soaking the cement specimens (10 mm × 10 mm × 70 mm) in water. Weight measurements were taken every 15 min until constant weight, meaning that the specimens were unable to absorb more water. Finally, immersed mass was determined on a hydrostatic scale. Volume was calculated following Equation (2) and apparent density, according to Equation (3).

(2) $$V=\frac{m_{sat}-m_{im}}{\rho_{w}},$$

where $m_{sat}$ is the saturated mass after specimens soaking in water, $m_{im}$ is the immersed mass of the specimens and $\rho_{w}$ is the density of water.

(3) $$\rho_{a}=\frac{m_{dry}}{V}$$

where $\rho_{a}$ is apparent density, $m_{dry}$ is the dry mass of the specimen and *V* is the above calculated volume.

The distribution of the LCMNF was observed in already-tested specimens through Field Emission Scanning Electro N·m icroscopy (FE-SEM) techniques, by means of a Hitachi S-3000 (Hitachi Europe S.A., Krefeld, Germany) working at 12 kV; samples were previously covered with carbon via sputtering.

Flexural strength was determined according to UNE-EN 196-1. Specimens of 40 mm × 40 mm × 160 mm were used. Flexural strength was determined in a universal testing machine, equipped with the corresponding clamping jaws for each test. A three-point apparatus was set, where force was applied perpendicularly to the specimen. Mechanical testing was performed after 7, 14, and 28 days of composite curing. Five test specimens were prepared and tested in each case.

Durability of the resulting composites was assessed by means of immersing standard flexural specimens in water for three days. Composites were tested at flexural every twelve hours with the aim of determining the influence of water absorption on mechanical properties. Prior to immersion, the specimens had been curing for 28 days. 

## 5. Conclusions

In this paper, the effect of four different dosages of LCMNF on the flexural strength of cement mixtures was examined. The main conclusions are as follows:

- The flexural strength of all the dosages analyzed shows that samples improved their flexural strength when the amount of LCMNF was increased. The most important increase occurred between 1.0 and 1.5 wt% at 28 days curing;
- FE-SEM micrographs for reinforced cement with 1.5 wt% CMNF showed the right bond formation between LCMNF and the hydration product;
- A slight reduction in the flexural strength of the mixtures with LCMNF was observed after immersion time in water during 72 h;
- The use of LCMNF substantially increased flexural strength if we compare with the control sample and with other cement composites made with processed or unprocessed natural fibers.

## Figures and Tables

**Table 1:** Chemical and morphological analysis of the raw material and pulps.

Parameter	Sawdust	Unbleached Pulp	Bleached Pulp
Yield (%)	-	45.4	96.7
Kappa number	88	25	20
Klason lignin (%)	30.61	14.72	11.7
Extractives (%)	2.04	0.80	0.64
Hemicellulose (%)	12.30	5.89	5.51
Cellulose (%)	55.05	78.62	82.15
Length ^1^ (µm)	-	576	572
Diameter (µm)	-	24.1	23.9
Fines ^2^ (%)	-	67.4	68.9

^1^ Weighted in length; ^2^ Fibers shorter than 75 µm.

**Table 2:** Characterization of the obtained lignocellulosic micro/nanofibers (LCMNF).

Yield of Fibrillation (%)	Transmittance at 800 nm (%)	CC (µeq-g/g)	CD (µeq-g/g)	σ (m^2^/g)	Diameter (nm)	DP
21.03	41.6	46.74	228.11	88.3	28	307

CC is carboxyl content; CD is cationic demand; σ is specific surface; DP is degree of polymerization.

**Table 3:** Experimental batch and doses of each material for a batch of 50 g.

Sample	LCMNF (wt %)	Cement (g)	Wet LCMNF (g)	Dry LCMNF (g)	Water (g)
C0	0.00	37.50	0.00	0.00	12.50
C0.5	0.50	37.31	2.34	0.19	10.34
C1.0	1.00	37.13	4.69	0.38	8.19
C1.5	1.50	36.94	7.03	0.56	6.03
C2.0	2.00	36.75	9.38	0.75	3.88
